# Organic Nanoplatforms for Iodinated Contrast Media in CT Imaging

**DOI:** 10.3390/molecules26237063

**Published:** 2021-11-23

**Authors:** Peng Zhang, Xinyu Ma, Ruiwei Guo, Zhanpeng Ye, Han Fu, Naikuan Fu, Zhigang Guo, Jianhua Zhang, Jing Zhang

**Affiliations:** 1 Department of Cardiology, Tianjin Chest Hospital, Tianjin University, Tianjin 300222, China; peng306588_0@163.com (P.Z.); 2019207009@tju.edu.cn (X.M.); cdrfnk@163.com (N.F.); zmedicalscience@163.com (Z.G.); 2 Key Laboratory of Systems Bioengineering of the Ministry of Education, Department of Polymer Science and Engineering, School of Chemical Engineering and Technology, Tianjin University, Tianjin 300350, China; rwguo@263.net (R.G.); yzphg@tju.edu.cn (Z.Y.); 3 Graduate School, Tianjin Medical University, Tianjin 300070, China; fuhan716@tmu.edu.cn; 4 Tianjin Key Laboratory of Membrane Science and Desalination Technology, Tianjin University, Tianjin 300350, China

**Keywords:** biomedical imaging, iodinated contrast media, X-ray computed tomography, organic nanoparticles, iodinated polymers

## Abstract

X-ray computed tomography (CT) imaging can produce three-dimensional and high-resolution anatomical images without invasion, which is extremely useful for disease diagnosis in the clinic. However, its applications are still severely limited by the intrinsic drawbacks of contrast media (mainly iodinated water-soluble molecules), such as rapid clearance, serious toxicity, inefficient targetability and poor sensitivity. Due to their high biocompatibility, flexibility in preparation and modification and simplicity for drug loading, organic nanoparticles (NPs), including liposomes, nanoemulsions, micelles, polymersomes, dendrimers, polymer conjugates and polymeric particles, have demonstrated tremendous potential for use in the efficient delivery of iodinated contrast media (ICMs). Herein, we comprehensively summarized the strategies and applications of organic NPs, especially polymer-based NPs, for the delivery of ICMs in CT imaging. We mainly focused on the use of polymeric nanoplatforms to prolong circulation time, reduce toxicity and enhance the targetability of ICMs. The emergence of some new technologies, such as theragnostic NPs and multimodal imaging and their clinical translations, are also discussed.

## 1. Introduction

Noninvasive in vivo bioimaging techniques are extremely valuable and useful for the visualization of an abnormal state within the body, the detection of the pathological situations of patients, assessment of the therapy efficacy and disease management [1,2]. According to the energy or signal sources to produce images, imaging modalities in the clinic are generally categorized using the following: ultrasound imaging (US), photoacoustic imaging (PA), positron emission tomography (PET), single-photon emission computed tomography (SPECT), fluorescence imaging (FI), luminescence imaging (LI), nuclear magnetic resonance imaging (MRI) and X-ray computed tomography (CT) [3]. Each imaging modality possesses its own unique advantages along with intrinsic limitations. US imaging offers real-time noninvasive imaging of soft tissue based on high-frequency sound waves. Its advantages include the fact that it is highly portable, has a low cost and is free of radiation risk, but its clinical applications are not suitable for adipose tissues and bones, and it can be strongly interfered with by air or gas and air-filled tissues [4]. PET and SPECT as high-resolution imaging modalities still suffer from the health hazard of radioactive components and extremely high costs [5]. The advantages of FI and LI imaging are their high sensitivity and high temporal resolution. Nevertheless, their clinical applications are severely impeded by the limited depth of light penetration through the tissues [6]. As a radiation-free and safe medical imaging technique, MRI can provide anatomical images of soft tissues, organs and blood vessels, but this expensive modality can be easily distorted by metal objects in the body [7].

Among all imaging modalities, computed tomography (CT) imaging has become one of the most powerful and popular imaging modalities for diseases diagnosis in modern clinical practice [1,8,9,10]. It can offer three-dimensional (3D) anatomic images with excellent spatial resolution based on X-ray attenuation. However, CT imaging can only offer superior images of electron-dense materials. To achieve high contrast within the body, it needs a very large difference between atomic weights or material densities within the patient. For example, due to the big difference between electron-dense bones and surrounding soft tissues, bone structures in the whole body can be visible using CT imaging under X-ray irradiation. However, for soft tissues with similar densities, their exquisite details cannot be distinguished clearly using CT imaging. Additionally, thus, to clearly delineate various tissues and detect subtle changes within tissues, the administration of exogenous contrast media is often required for most patients for effective CT imaging. Exogenous CT contrast media are distributed into different tissues, affording transient contrast enhancement in soft tissues under X-ray irradiation.

In current clinical practice, barium- and iodine-based compounds are routinely used as contrast media for in vivo CT imaging. Barium-based contrast media are restricted only to gastrointestinal tract imaging via oral route due to their inherent high toxicity. Therefore, iodinated contrast media (ICMs) have become the most prevalent intravenous media used for X-ray CT imaging. The yearly use of the ICMs was estimated to reach approximately 90 million doses worldwide.

## 2. Iodinated Contrast Media

**Figure 1:**
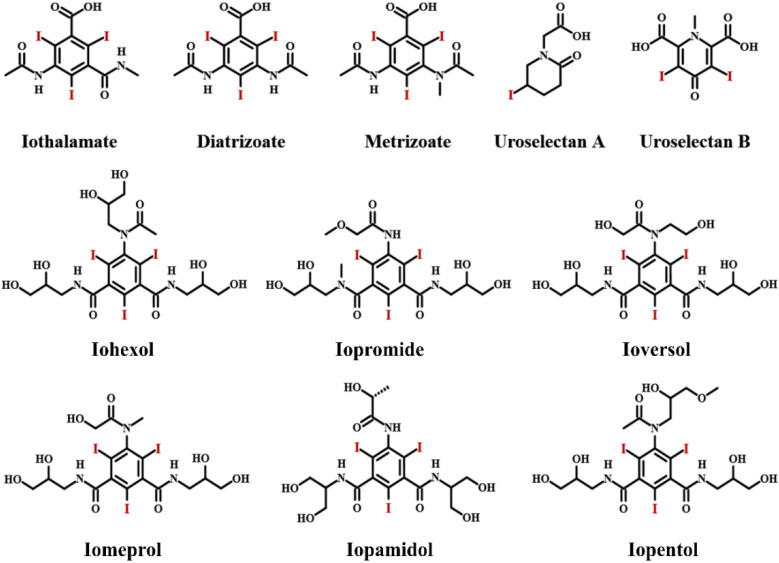
Ionic and nonionic iodinated contrast media.

For better visualization of soft tissues and especially for identifying the interface between two adjacent soft tissues, the presence of a great difference in X-ray attenuation around the lesion location is indispensable for CT imaging to achieve high contrast-to-noise ratios. The contrast media containing high-Z elements can enhance differentiation among different tissues, because the X-ray attenuation effect of a material generally increases with its atomic number [9,11]. Iodine has historically been the atom of choice for the applications of CT imaging, and now, small iodinated compounds predominantly dominate X-ray contrast media due to their high atomic number (Z = 53), high X-ray absorption coefficient and their great flexibility and versatility in chemical synthesis [12]. Water-soluble sodium iodide and potassium iodide are among the earliest contrast media, first used in 1924. However, at the concentrations necessary for imaging, inorganic iodine solutions exhibit high toxicity, which severely hinder their clinical applications. The development of ICMs rapidly moved from inorganic iodine to organic iodinated molecules. Some commercially available, clinically approved organic ICMs are summarized in Figure 1. Organic ICMs started as ionic mono-iodinated, di-iodinated and tri-iodinated molecules. As shown in Figure 1, ionic iodinated molecules mainly include Iothalamate, Uroselectan A, Uroselectan B and Diatrizoate, which are often the derivatives of iodine-containing benzoic acid [8,9,13,14]. These ionic iodinated molecules are high osmolar contrast materials, which are associated with some severe side effects.

**Figure 2:**
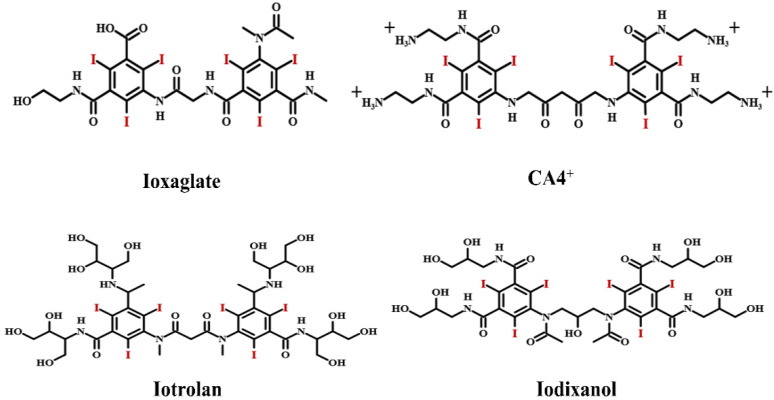
Ionic and nonionic dimers as iodinated contrast media.

Continuous efforts were directed toward minimizing risks of contrast reactions during the 1960s. Compared with ionic molecules, nonionic compounds do not dissociate in water and thus have much lower osmolality. Moreover, they have a lower tendency to interact with cell membranes, peptides and other biological structures. Therefore, the toxicity of nonionic compounds is significantly lower than that of ionic molecules. As a result, the exploitation of ICMs with improved imaging capabilities and reduced toxicity focused on the nonionic iodinated molecules. As presented in Figure 1, the nonionic iodinated contrast media mainly include Iohexol, Iopromide, Ioversol, Iomeprol, Iopamidol and Iopentol. Nearly all of them are tri-iodinated benzene derivatives with very similar structures. The benzene ring not only can offer a stable framework for the three adjacent iodine atoms, but also increase the effective molecular size and decrease the toxicity. Moreover, the other positions of the benzene ring are occupied by long side chains rich in hydroxy groups, ensuring high water solubility and low toxicity. In addition to iodinated monomers, ionic and nonionic dimers were also developed, such as Ioxaglate, Iotrolan and Iotrolan, as shown in Figure 2. Compared with monomers, dimers possess much lower osmolality, which can alleviate pain at the site of injection and decrease the renal injury and cardiovascular complications. However, the viscosity of nonionic dimers is much higher than that of monomers, often leading to a slower excretion and more painful injection compared with some monomeric agents [8,9,13,14].

## 3. Iodinated Macromolecular Contrast Media

**Figure 3:**
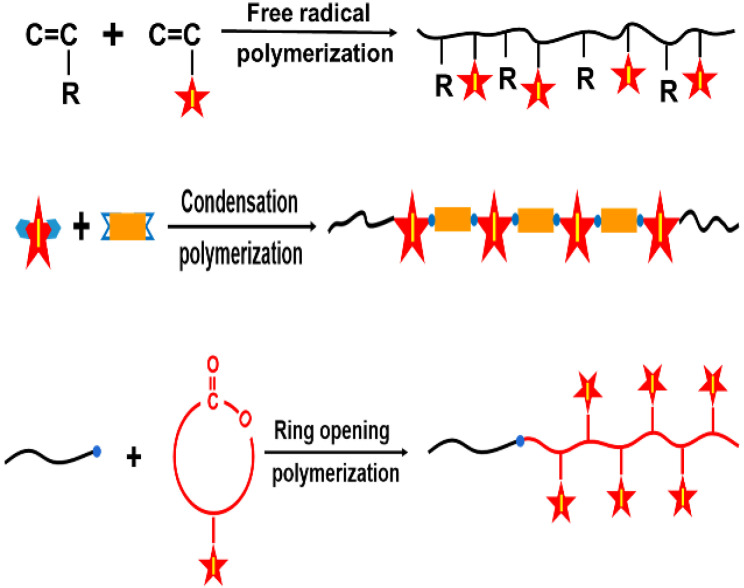
Polymerization strategies for combining polymer and ICMs to prepare iodinated macromolecular contrast media via free radical polymerization, condensation polymerization and ring opening polymerization of iodine-containing monomers, (Star refers to iodine-carrying groups or compounds).

The last several decades have witnessed tremendous progress in nonionic ICMs. However, their clinical applications are still hampered by some limitations: (1) rapid renal excretion and thus a very short circulation time [10,14,15]; (2) serious adverse effects, especially contrast-induced nephropathy (CIN), with potential life-threatening injuries [16,17]; (3) inefficient targetability and thus unclear CT imaging at target lesions [18,19]. In order to address these limitations, great efforts have been made to develop and optimize these small-molecule ICMs [8,9,20,21,22,23]. Due to their unique biocompatibility, designability, biodegradation, facile synthesis and modification capability, polymers have opened up a new avenue to enhance the delivery efficacy and biocompatibility of ICMs [24,25,26,27,28,29,30,31,32,33,34,35]. The applications of polymers for ICM delivery can be achieved using a combination of polymers and small iodinated compounds through various mechanisms. One of the most important approaches is the design and preparation of iodinated macromolecular contrast media. The main strategies based on polymerization technologies for combining polymer and ICMs to prepare macromolecular contrast media are shown in Figure 3. The macromolecular contrast media can be prepared by free radical polymerization, condensation polymerization or ring-opening polymerization of iodine-containing monomers [36,37,38,39]. For example, triiodobenzoate-containing vinylic monomers, such as 3-(methacryloy-lamidoacetamido)-2,4,6-triiodobenzoic acid (MABTIB) [40], 2-hydroxy-3-methacryloyloxypropyl (2,3,5-triiodobenzoate) (HMTIB) [41,42] and 2-methacryloyloxyethyl (2,3,5-triiodobenzoate) (MAOETIB) [36,43] were widely used to prepare radiopaque polymers by homo-polymerization or copolymerization with other vinylic monomers. It is worth pointing out that some iodine-containing vinylic monomers, such as triiodophenyl methacrylate, only can be used to obtain low molecular weight polymers (oligomers), due to the steric hinderance effect of the iodinated aromatic nucleus [44]. In addition, some iodine-containing diol monomers, such as 2,2-bis(iodomethyl)-1,3-propanediol [38] and 2,2-bis(hydroxymethyl)propane-1,3-diyl bis(2,3,5-triiodobenzoate) [45], were used to undergo condensation polymerization with diacids to prepare iodinated polyesters as a versatile platform for radiopaque biomaterials. Moreover, ring-opening polymerization also can be applied to prepare iodinated macromolecular contrast media. For example, a new iodine-functionalized trimethylene carbonate as monomer can be used in ring-opening polymerization using CH_3_O-PEG-OH as an initiator and zinc bis[bis(trimethylsilyl) amide] as a catalyst to prepare iodinated polymer poly(ethylene glycol)-b-poly(iodine trimethylene carbonate) with an ultrahigh iodine content of 60.4 wt.% [30].

**Figure 4:**
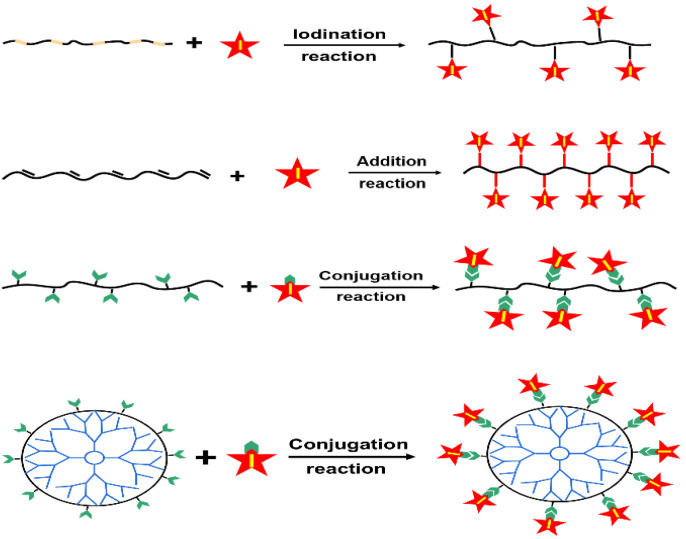
Reaction strategies for combining polymer and ICMs to prepare iodinated macromolecular contrast media via iodination reaction, iodine addition reaction and chemical conjugation of ICMs on polymer chains, the surface of dendrimers or hyperbranched polyesters, (Star refers to iodine-carrying groups or compounds).

In addition, iodinated macromolecular contrast media can be also prepared via the modification or functionalization of polymer chains via iodination reaction, addition reaction and conjugation or graft reaction [46,47,48], as summarized in Figure 4. For example, polyvinyl phenol can be iodinated via aromatic electrophilic substitution, using sodium iodide (NaI) as an iodination reagent [46]. Iodic acid (HIO_3_) was also used as an iodination reagent to prepare iodinated macromolecular contrast media [48]. In addition, the addition reaction between iodine and unsaturated carbon compounds was widely used as an effective approach to prepare diiodine compounds and iodinated polymers. For example, iodinated chitosan derivatives were prepared using the iodine addition reaction [49]. The most widely used strategy for the synthesis of iodinated polymers is chemical conjugation reaction. The chemical conjugation of iodinated compounds onto polymer backbones [50,51,52,53] or onto the surface of polymers including dendrimers [54,55,56] and star polyesters [45,57] was also widely used to prepare various macromolecular contrast media. Due to the high simplicity and versatility in preparation, the wide availability of starting materials and the extremely high reactivity with alcohols and phenols, or ammonia and amines, iodine-containing acyl chlorides (especially for 2,3,5-triiodobezoyl chloride) were widely conjugated onto various polymer chains, such as celluloses [58], chitosan [50,59] and polyvinyl alcohol [52], as well as dendrimers [54,55,56]. These results indicated that strategies based on iodinated macromolecular contrast media have great potential to overcome those intrinsic limitations of small molecular ICM compounds. In addition, it is worth pointing out that iodinated macromolecular contrast media often suffer from a relatively low iodine content. Nonetheless, macromolecular contrast media with tailored functionality have opened up new possibilities for precise imaging and diagnosis.

Apparently, the strategies and applications of polymers to produce iodinated macromolecular contrast media can endow the unique advantages of polymers to small molecular ICMs, especially the ability of self-assembly into nanostructures, which will open a new avenue for the future design of biosafe and efficient CT contrast media. Moreover, both small molecular ICMs and iodinated macromolecular contrast media can also be loaded into various nanocarriers to form nanoscale contrast media, which can thoroughly improve the efficacy of ICMs’ delivery and change their metabolic pathway, thus exhibiting great potential to address the abovementioned issues of traditional ICMs [1,8,20,60,61,62,63,64]. Nevertheless, an exhaustive discussion on the biomedical applications of organic nanoparticles (NPs) for delivering ICMs is currently missing in the literature. Herein we comprehensively summarize the strategies and applications of organic NPs, including liposomes, nanoemulsions, micelles, polymersomes, dendrimers, polymer conjugates and polymeric particles, for the efficient delivery of small molecular ICMs and iodinated macromolecular contrast media. We mainly focus on the use of polymeric nanoplatforms to prolong circulation time, reduce toxicity and enhance the targetability of ICMs. The emergence of some new technologies, such as theragnostic NPs and multimodal imaging and their clinical translations, are also discussed.

## 4. Organic Nanoparticles for ICMs Delivery

### 4.1. Nanoparticles for Biomedical Applications

**Figure 5:**
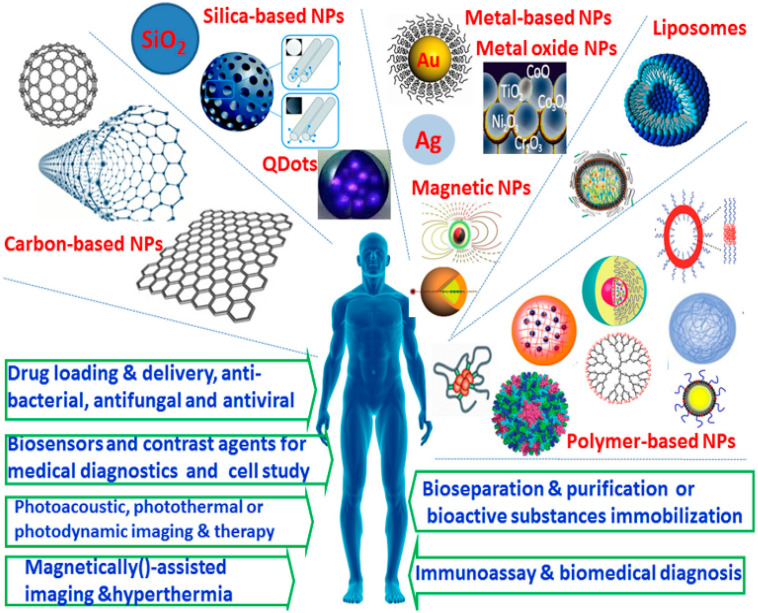
Types of nanoplatforms currently described in biomedical fields [65]. Reprinted with permission from ref. [65], Copyright 2015 MDPI.

In the past several decades, nanoparticulate systems have gained a great amount of attention as one of the most promising biomedical materials, due to their unique physicochemical properties, nano-sized characteristics, controlled shape and versatile modification possibilities, as well as well-defined multifunctionalities. A wide variety of nanomaterials, such as carbon-based NPs, silica-based and other inorganic NPs, semiconductor NPs, metal and metal oxide NPs, as well as organic NPs (e.g., liposomes, nanoemulsions and polymer-based NPs, including micelles and nanogels, polymersomes, dendrimer, polymer-drug conjugations and protein NPs) have been developed and employed in a diverse array of biomedical fields, as shown in Figure 5. These nanomaterials provide a powerful platform for the site-specific and controllable delivery of drugs, genes, proteins and contrast agents; some of them exhibit noticeable antibacterial, antiviral and antifungal activities. Some inorganic and metal NPs with unique physicochemical properties can be used for photoacoustic, photothermal or photodynamic as well as hyperthermal therapy. In addition, some functional NPs can find wide applications in a new generation of intelligent biosensing, bioseparation, cell labeling, bioimaging and diagnosing.

The in vivo transportation behavior and metabolic processes of NPs are different from traditional small molecular compounds. After invading a biological milieu, NPs will inevitably make contact with a huge variety of biomolecules in body fluids or blood, such as sugars, proteins and lipids, leading to the formation of the so-called “protein corona” and clearance via the reticuloendothelial system (RES) and/or mononuclear phagocytic system (MPS). Undoubtedly, the circulation behavior and time in blood of NPs are critical for their biodistribution and metabolism, accumulation in targeted tissues and thus therapeutic and diagnostic efficacy. As is well known, the in vivo behaviors of NPs are dictated by their physicochemical properties, such as hydrophilic–lipophilic properties, surface feature and surface charge, particle size and particle shape. For example, the hydrophilicity of NPs can impede aggregation and opsonization in water or serum and prolong the circulation time of NPs. Surface charge is another important factor that can definitely affect the fate of NPs administered in biological systems. Positively charged NPs have higher affinity with negatively charged cell membrane but often suffer from serious aggregation and rapid clearance after injection due to nonspecific interactions with blood components. The size and shape of NPs also contribute significantly to their biodistribution in circulation and interaction with tissues and cells. Generally, the ideal size of NPs for long circulation is in the range of 20–200 nm. The size of NPs should be larger than 20 nm in diameter in order to avoid filtration via the kidney and smaller than 200 nm to avoid specific sequestration via fenestra of liver and sinusoids in spleen. Spherical NPs can be more efficiently taken up by cells than non-spherical NPs with similar sizes and under the same conditions. Nevertheless, non-spherical NPs exhibit superior properties to their spherical counterparts in terms of escaping from phagocytosis and circulating in blood. In sum, the effect of the physicochemical properties of NPs on biological systems is very complicated and unclear, which should be fully demonstrated prior to the widespread application of NPs in pharmaceutical, biomedical and diagnostic fields.

As mentioned above, iodine-based contrast media have shown a very high potential for CT diagnostic applications. However, some inherent drawbacks, such as the short circulation time, poor biocompatibility and inefficient targeting capability, inhibited the more widespread application of such media. To meet increasingly rigorous requirements for clinical use, a great number of approaches have been explored to effectively surmount these drawbacks [8,9,21,22,23,61,62,66,67]. The strategies and applications of organic NPs with desirable functions and excellent performances for medical imaging have gained an enormous amount of attention [3,32,33,34,38,68,69,70,71,72,73,74,75,76]. This is due to the fact that organic NPs possess a great number of desirable physicochemical properties, such as simplicity for drug loading, high biocompatibility, desirable biodegradation, facile synthesis, low cost as well as great flexibility and versatility in modification or functionalization [28,34,61,62,65,72,73,74,75,76,77,78,79,80,81,82]. Apparently, the applications of organic NPs and especially polymeric NPs for ICMs’ delivery offer an excellent improvement in X-ray imaging and medical diagnosis [33,34,83]. A variety of organic NPs, such as PEGylated liposomes, nanoemulsions, micelles, polymersomes, dendrimers and natural NPs, have been explored in the development of functionalized contrast media with better biocompatibility, longer circulation time or more efficient targeting capability [8,33,34,64,76,83]. Different types of organic NPs for ICMs’ delivery are described below.

### 4.2. Liposomes for ICMs Delivery

**Figure 6:**
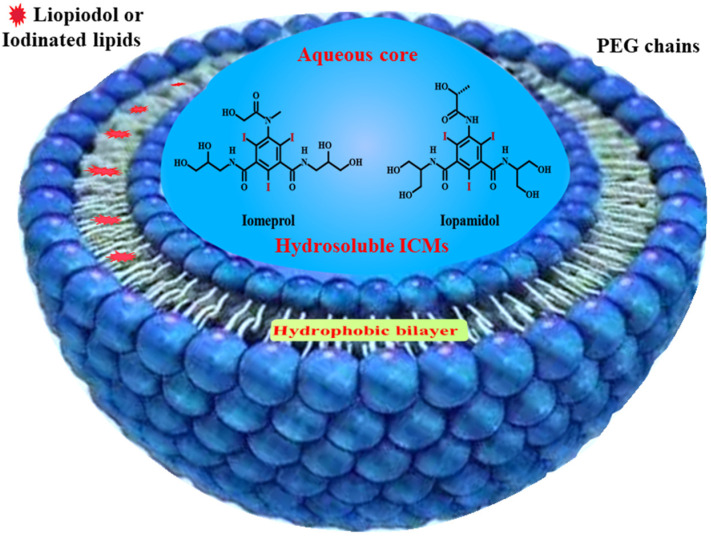
Liposomes structure for ICMs’ delivery. Hydrophobic ICMs loaded within bilayer; hydrosoluble ICMs encapsulated in aqueous core.

As mentioned above, small iodinated compounds are widely used as injectable CT contrast media in the clinic. However, because small ICMs can be rapidly cleared from the bloodstream through the kidney, one of the long-standing challenges for their application is their inherently short circulation time, leading to a very narrow window for imaging after injection and serious side effects in the excretion pathway [8,10,60]. Great effort has been made to develop long-acting forms of ICMs. As one of the earliest and most widespread nanotechnologies for drug delivery, liposomes have been widely used for ICMs’ delivery [82,84,85,86,87]. As shown in Figure 6, liposomes consist of an aqueous core enclosed by a lipid bilayer of natural phospholipids, which can be used to encapsulate hydrosoluble ICMs such as Iopamidol and Iodixanol in an aqueous core and load iodinated oils within the hydrophobic bilayer [88]. Due to their high biocompatibility from the innocuous nature of phospholipids, the first batch of liposomal formulations containing contrast agents was reported in the 1980s, which achieved enhanced vascular and hepatic imaging [89,90,91]. However, these liposomes can be rapidly recognized and cleaned by the immune system.

Some hydrophilic polymers have been proved to be able to prolong the blood circulation time of drug delivery systems. Among them, polyethylene glycol (PEG) and PEG-containing polymers are the most commonly employed in biological and pharmaceutical fields for the development of long-circulating drug delivery systems [92,93,94]. This is due to the fact that the PEG chain provides a high hydration level, big hydrodynamic size and strong steric hindrance, which can not only enhance in vivo stability, but also prevent the attachment of serum proteins and impede uptake by the reticuloendothelial system, leading to a significant decrease in the clearance rate from circulation. As a result, the modification of liposomal surfaces with PEG was widely applied to formulate long-circulating liposomes with improved pharmacodynamic properties for a variety of pharmaceutical, biomedical and bioimaging applications. For example, PEG phospholipids, such as 1, 2-distearoyl-sn-glycero-3-phosphoethanolamine-poly(ethylene glycol) (DSPE-PEG), were widely used and successfully demonstrated to be able to enhance stability, improve encapsulation efficiency and prolong the blood circulation time of liposomes [95,96]. Water-soluble ICMs, such as Iohexol [97] or Iodixanol [98], were encapsulated into the core of PEG-coated liposomes as effective blood pool contrast media for use in long-term imaging of pulmonary arteries. Iodixanol-loaded liposomes can maintain contrast enhancement over several hours in rabbits. On the contrary, Iodixanol is rapidly cleared from the body within minutes [98]. Moreover, renal filtration was found to be a non-dominant approach for liposome clearance from blood, which can decrease the risk of contrast-induced nephrotoxicity.

In addition to rapid clearance via kidney, the inefficient targetability of ICMs delivery is another key issue yet to be resolved, as small molecular ICMs are nonspecific compounds. After intravascular injection, they are mainly distributed within the extracellular fluid compartment and then rapidly cleared from human blood via kidney filtration and clearance, which not only results in a short timeframe for CT imaging and undesirable renal toxicity, but also impedes the specific visualization and detection of target tissues [14,99,100]. Delivery systems for liposomal ICMs have attracted significant research interest and demonstrated targeted imaging of tumor tissues, cardiovascular diseases and lymph nodes, as liposomes can deliver both drug molecules and contrast media via passive targeting [14]. Generally, one of the main mechanisms of passive delivery is the reticuloendothelial system (RES)—recognized accumulation [101,102,103]. After intravenous injection, exogenous nanoparticles are rapidly recognized and sequestered by RES and hepatocytes in spleen and liver, especially for non-PEGylated (i.e., non-stealth) nanoparticulate drug delivery systems. In addition, even PEGylated long-circulating nanoparticles can also gradually accumulate in the liver via RES and hepatocytes. This kind of phenomena has initiated a surge of development in the passively targeted delivery of X-ray contrast media to spleen and liver. For example, some studies have demonstrated that the non-PEGylated iodinated liposomes specifically accumulated in the spleen and liver [104,105]. For example, Kweon et al. reported a kind of liposome that simultaneously loaded water-soluble iodinated compound (Iopamidol) and an iodinated ethyl ester of poppy seed oil (Lipiodol) via the modified reverse-phase evaporation method. Compared with free liposomes or liposomes loaded with Iopamidol alone, liposomes coloaded with Iopamidol/Lipiodol after intravenous injection into rats produced more pronounced contrast enhancement and more significant persistence in RES-rich organs, such as the liver and spleen. These results indicated that liposomes can serve as an RES-targeted contrast agent for targeted CT imaging [106].

The other main mechanism of passive delivery is the accumulation of delivery systems for nanoparticulate ICMs through the enhanced permeation and retention (EPR) effect. The EPR effect is a property where there is a significantly higher accumulation of macromolecules and nanoparticles with appropriate nanoscale size in tumor tissues than in normal tissues [107,108,109]. In normal tissues, the tight junctions of endothelial cells prevent the transport of nanoparticles. In contrast, due to the leakage of tumor vasculature and poor lymphatic drainage, tumor tissues can selectively accumulate and retain macromolecular drugs and nanoparticles, especially for PEGylated long-circulating nanomedicines. Therefore, the EPR effect has become an important guiding principle for the development of nanomedicines and nanoparticulate contrast media for cancer treatment and diagnosis [18,85,95]. The Allen group tried to longitudinally quantify and visualize the biodistribution of Iohexol-containing PEGylated liposomes in various body compartment volumes over a 14-day period in VX2 sarcoma-bearing New Zealand White rabbits using volumetric high-resolution CT imaging [110]. The results indicated that liposomes can be passively accumulated at tumor sites through the EPR effect. Other PEGylated liposomes, including Iopamidol-loaded liposome [95] and Iodixanol-loaded liposome [111], also achieved a prolonged blood pool contrast enhancement and an increased accumulation of iodinated liposomes in tumor tissues via the EPR effect.

**Figure 7:**
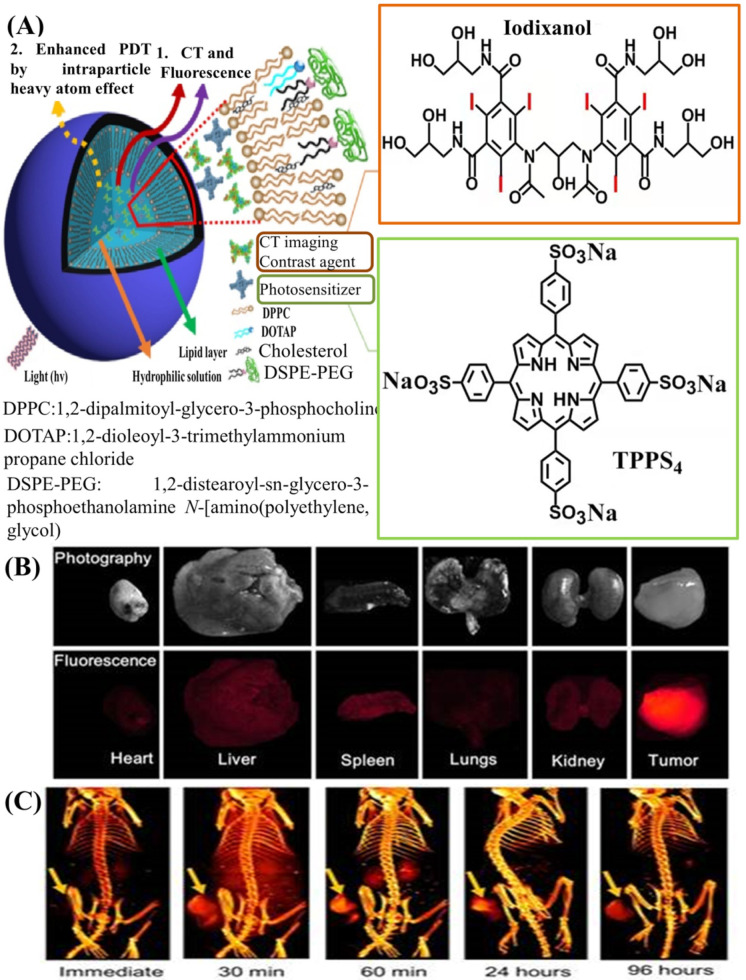
Liposomes co-encapsulating Iodixanol TPPS_4_ for concurrent CT and FL imaging [116]. (**A**) Schematic diagram illustrating the structure and composition of liposomes; (**B**) photography and fluorescence images of the major organs resected from mouse after 96 h injection; (**C**) CT images at different time after injection; Reprinted with permission from ref. [116], Copyright 2021 Ivyspring.Although liposomes, especially PEG-coated liposomes, used for ICMs delivery can prolong the blood circulation time, increase targetability via the EPR effect and enhance sensitivity via multimodal imaging, they still suffer from a variety of challenges. In addition to the complexity of the formulation processes and very low drug loading, the stability of liposomes is one of their main disadvantages. Liposomes can be degraded through various physicochemical processes, such as auto-oxidation, hydrolysis, destabilization by dilution, self-aggregation and coalescence, often leading to premature ICMs leakage during storage and a strong ICMs burst release in blood. More research from various fields is needed to truly exploit the clinical experience of liposomes in combination with ICMs toward more capable imaging.

It is well known that each imaging modality has its own unique advantages and intrinsic limitations. Imaging modalities with high resolution often suffer from relatively low sensitivity resolution, while those with high sensitivity have relatively poor resolution. Recently, to resolve this problem, the use of multimodal imaging, i.e., combining two or more imaging modalities into one system, has gained significant attention, because this synergistic method of imaging can overcome the limitations and take advantage of the strengths of each modality [1,112,113,114,115]. For example, imaging modalities with high spatial resolution (such as CT imaging) are frequently combined with other imaging modalities with high sensitivity (PET, optical, etc.). A complementary combination of CT imaging with high resolution and fluorescence (FL) imaging with high sensitivity has exhibited some desirable advantages in cancer diagnostics. Recently, Xu et al. reported a kind of PEGylated liposome that co-encapsulated clinically approved Iodixanol as ICMs and hydrophilic meso-tetrakis(4-sulphonatophenyl) porphine (TPPS_4_) as a photosensitizer for concurrent CT and FL imaging-guided cancer theragnostics [116], as shown in Figure 7A. Liposomes with sizes of about 100 nm were found to have an enhanced passive tumor uptake via the EPR effect, along with insignificant accumulation in the liver and other organs. Their highly tumor-specific biodistribution was manifested using both FL (Figure 7B) and CT imaging (Figure 7C), which can demonstrate the applicability of liposomes as contrast agents for bimodal tumor imaging and the imaging-guided treatment of cancer.

### 4.3. Nanoemulsions for ICMs Delivery

**Figure 8:**
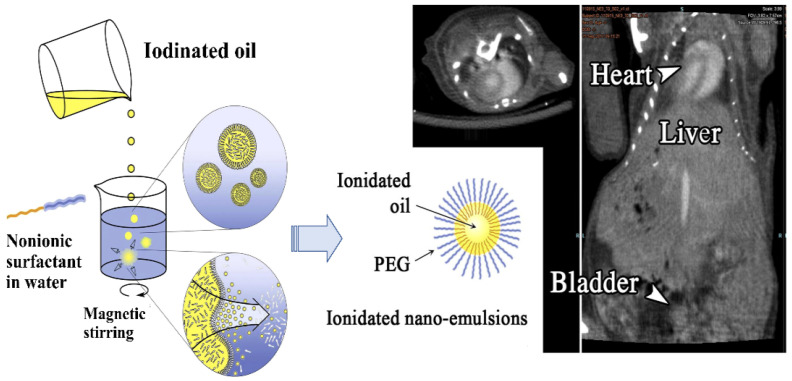
Schematic representation of the formation process of iodinated nanoemulsions and application for CT imaging [121]. Reprinted with permission from ref. [121], Copyright 2013 Elsevier.

In addition to liposomes, significant progress was recently observed in exploiting the ICMs-loaded nanoemulsions as an effective contrast agent with improved performance [117]. The nanoemulsions-based contrast media were generally a colloidal dispersion form of ICMs with diameters ranging from 20 to 200 nm. The ICMs-loaded nanoemulsions generally consisted of water-insoluble iodinated oil and different types of lipids as the oily phase and PEGylated surfactants or PEG-containing block polymers as dispersion stabilizers in an aqueous medium [118,119,120,121,122,123]. As shown in Figure 8, after mixing the organic iodinated oil into the aqueous solution of PEGylated nonionic surfactants, the lipophilic molecules in the form of nanoscale droplets were immediately stabilized by the surfactant molecules, leading to the formation of an iodinated oily core surrounded by a hairy layer of the PEG moiety from nonionic surfactant. For nanoemulsions, the dispersion stabilizers are very important to govern the phase behavior of nanoemulsions and inhibit the occurrence of the flocculation, coalescence and sedimentation of nanoemulsions. Moreover, the presence of free surfactants had a significant impact in regard to the elimination, pharmacokinetics and biodistribution of nanoemulsions [121].

Some ICMs-loaded nanoemulsions are commercially available, such as Fenestra^®^, which consists of poly-iodinated triglyceride (ITG) and phospholipids and cholesterol as a dispersion stabilizer [124]. These iodinated nanoemulsions are mainly used for blood pool or liver/spleen preclinical imaging and found an important place in the market of preclinical CT contrast agents. However, their iodine content is relatively low and thus the injection of a relatively large volume must be required, often leading to a non-negligible toxicity of the product. To decrease the toxicity even more, Attia et al. used the PEGylated nonionic surfactant, PEG-35 castor oil (trade name Kolliphor^®^ ELP), to develop PEGylated nanoemulsions with iodinated monoglyceride and iodinated castor oil [118]. The obtained PEGylated nanoemulsions not only were endowed with very high iodine concentration, leading to a very strong X-ray attenuation property, but also achieved a very high contrast enhancement in blood with a half-life around 6 h. In addition, PEG-containing block polymers were demonstrated to be able to more effectively stabilize the nanoemulsions. Vries et al. synthesized three hydrophobic iodinated oils for use as the oily phase, based on the 2,3,5-triiodobenzoate moiety [119]. These new iodinated oils have a very high iodine content over 50%. In addition, then, poly(ethylene glycol)-b-poly(propylene glycol)-b-poly(ethylene glycol)(PEG-PPG-PEG, Pluronic F68) and poly(butadiene)-b-poly(ethylene glycol) (PBD-PEG) were used as dispersion stabilizers to prepare long-circulating blood pool contrast media. Compared with the commercial formulations (Fenestra^®^), the PBD-PEG stabilized nanoemulsions could exhibit much lower in vivo toxicity and achieved a longer blood circulation time, exhibiting great potential for use as blood pool agents in contrast-enhanced CT imaging. In addition, due to their excellent biocompatibility and biodegradability, a PEG-polyester, such as diblock copolymer poly(ethylene glycol)-b-polycaprolactone (PEG-PCL), was also used as a dispersion stabilizer to prepare ICMs-loaded nanoemulsions [125].

Compared with liposomal formulations, nanoemulsions actually have many more advantages. Firstly, the stability of nanoemulsions is much higher. They are relatively stable against dilution and under heating. In addition, their formulation and fabrication are much simpler and cheaper, especially for the fabrication process of nanoemulsion droplets. Moreover, nanoemulsions exhibited higher encapsulation efficacy of ICMs and had a higher loading capacity for water-insoluble ICMs or hydrophobic drugs than liposomes. Finally, PEG or specific ligands can be more easily introduced onto the droplets’ surface, conferring them strong stealth properties or targetability [88,124]. Apparently, nanoemulsions and liposomes have exhibited great potential for efficient ICMs delivery. However, these nanocarriers still suffer from the leakage of internal payloads and very low drug loading capacity.

### 4.4. Polymeric Nanoparticles for ICMs Delivery

**Figure 9:**
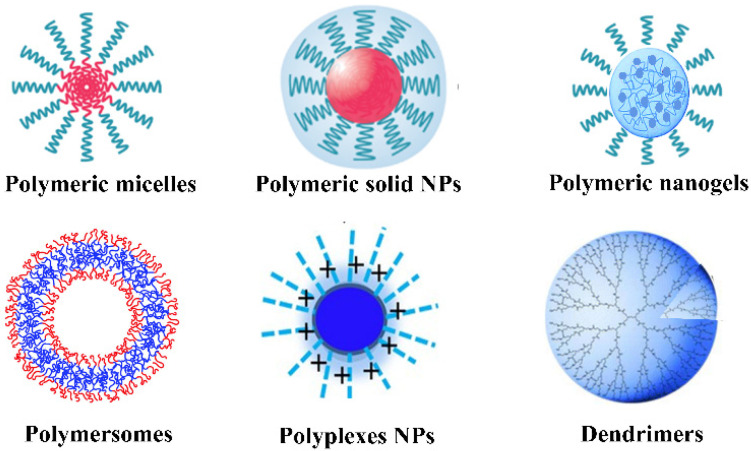
Schematic illustration of typical polymeric nanoparticles.

Over the past several decades, polymers and their related nanomaterials have gained great attention and exhibited great potential for biomedical and pharmaceutical applications due to their unique biocompatibility, designability, biodegradation, facile synthesis and modification capability. According to their morphology and composition in the core and periphery, polymeric NPs can be mainly categorized as micelles, solid NPs, nanogels, polymersomes, polyplexes and dendrimers, as shown in Figure 9. These polymeric NPs can be incorporated with drugs or ICMs via encapsulation or conjugation, which have opened up a new avenue to improve the biocompatibility, delivery efficacy and diagnosis sensitivity of ICMs [24,25,26,27,28,29,30,31,32,33,34,35].

#### 4.4.1. Polymeric Micelles

Polymeric micelles, a kind of aggregation colloid derived from the self-assembly of amphiphilic polymers in water, have been demonstrated as an effective approach to address the issues related to the delivery and release of drug or diagnostic agents [32,33,86,126]. Micelles are a kind of unique core–shell nanostructure. Their hydrophilic shells are mainly composed of PEG or similar hydrophilic polymers, such as polyvinyl pyrrolidone (PVP), polyvinyl alcohol (PVA), dextran, chitosan, hyaluronic acid, polyacrylic acid (PAA) as well as polyelectrolytes and zwitterionic polymers. Hydrophilic polymers on the shells provide stability to the cores in water. The hydrophobic cores are generally derived from the aggregations of hydrophobic chains or the co-assembly of lipophilic chains and hydrophobic drugs due to the hydrophobic interactions. Due to their excellent biocompatibility and biodegradability, polyesters, including poly(caprolactone) (PCL), poly(lactide) (PLA), poly(lactide-co-glycolide) (PLGA) and poly(amino acids), were widely accepted as hydrophobic polymers for the construction of micelles for pharmaceutical applications. The hydrophilic and hydrophobic chains can be easily tailor-made by changing the number of structural repeating units and the chain composition in each polymeric chain.

**Figure 10:**
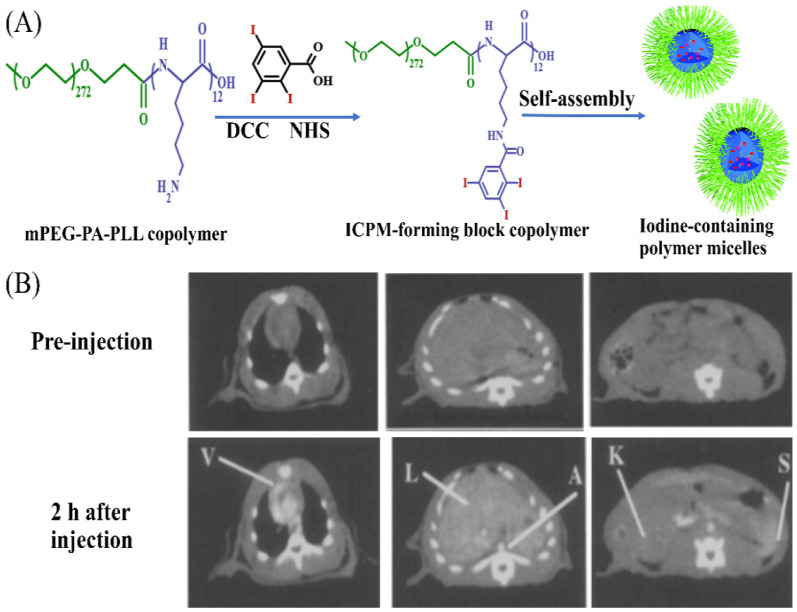
Micelles formed from iodinated amphiphilic block copolymer for CT imaging. (**A**) Schematic depiction of preparation process and chemical structure of ICPM-forming copolymer. (**B**) Micelles formed from ICPM-forming copolymer for CT imaging of a rat [126]. Reprinted with permission from ref. [126], Copyright 2013 Wiley.

The superior flexibilities in structure, composition and functionalization made polymeric micelles attractive for their use in of drug delivery, especially for hydrophobic drugs. Generally, drugs can be loaded in polymeric micelles via physical encapsulation to prepare polymeric drug delivery systems. However, it was ineffective and very difficult for common micelles to encapsulate ICMs via hydrophobic interactions. On the one hand, ICMs are typically water-soluble ionic or nonionic iodinated molecules, or water-insoluble iodinated oil. On the other hand, different from liposomes with an internal aqueous core and nanoemulsions with an internal oily core, polymeric micelles with aggregation-forming hydrophobic cores were not suitable for loading water-soluble molecules or lipophilic oil. Therefore, one of the main strategies for the fabrication of iodinated micelles is via the covalent linkage of ICMs to the hydrophobic tails of amphiphiles, and then self-assembly of the iodinated moieties into the center of the micelles. For example, the Torchilin group has developed a kind of amphiphilic block copolymer (mPEG-PA-PLL) by conjugating a hydrophilic PEG chain with 12-repeat-unit polylysine. Then, 2,4,6-triiodobenzoic acid was conjugated to the amine groups of the mPEG-PA-PLL chain to prepare iodinated amphiphilic block copolymer (ICPM-forming copolymer) [34,53], as shown in Figure 10A. The ICPM-forming copolymer possessed the ability of forming polymeric micelles with iodine content of about 17.7% by weight. A strong contrast of over 50 HU could still be observed in the heart, liver and spleen after injection in rat for 2 h (Figure 10B). This kind of iodinated polymeric micelle exhibited great potential for long-lasting blood pool contrast [34,53,124].

**Figure 11:**
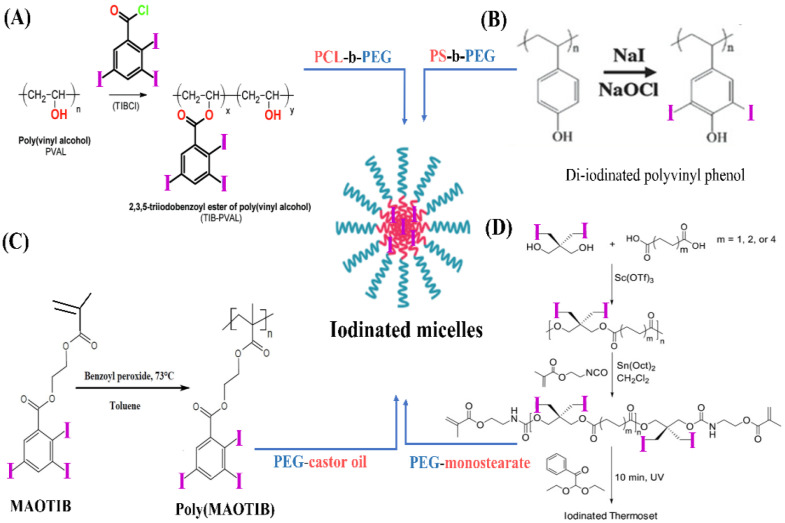
Iodinated macromolecular contrast agents for construction of micelles by co-assembly for CT imaging. (**A**) TIB-PVAL co-assembly with PCL-b-PEG [52]; Reprinted with permission from ref. [52], Copyright 2020 Royal Society of Chemistry; (**B**) di-iodinated polyvinyl phenol co-assembly with PS-b-PEG [46]; Reprinted with permission from ref. [46] Copyright 2018 Wiley; (**C**) poly(MAOTIB) co-assembly with PEG-35 castor oil [27]; Reprinted with permission from ref. [27], Copyright 2017 Elsevier; (**D**) iodinated thermoset co-assembly with PEG-monostearate [38]. Reprinted with permission from ref. [38], Copyright 2017 Wiley.

In addition, based on the self-assembly of amphiphilic polymers into micelles, amphiphilic macromolecular ICMs (PEG-PHEMA-I) were prepared via the atom transfer radical polymerization (ATRP) polymerization of 2-hydroxyethyl methacrylate (HEMA) in the presence of macro-ATRP initiator (PEG-Br) and subsequent esterification reaction with 2,3,5-triiodobenzoic acid to introduce iodine onto the side chain of PHEMA [127]. The obtained PEG-PHEMA-I can self-assemble into iodinated polymeric micelles. Moreover, to overcome the barriers of encapsulating ICMs into micelles, co-assembly between iodinated polymers and amphiphilic polymers was also widely used to prepare iodinated micelles. In this strategy, iodinated macromolecular contrast agents were prepared firstly as shown in Figure 3 and Figure 4, and then iodinated macromolecular contrast media were used as a building block to co-assemble with amphiphilic polymers or surfactant to form micelles, as shown in Figure 11. For example, Balegamire et al. prepared a kind of iodinated polymer (TIB-PVAL) via the attachment of tri-iodobenzoyl to the PVAL backbone. Then, TIB-PVAL was co-assembled with poly(caprolactone)-b-poly(ethylene glycol) (PCL-b-PEG) to form polymeric micelles with diameters of about 150 nm (Figure 11A). The intravenous injection of polymeric micelles into rats resulted in a clear visualization of the cardiovascular system over several hours [52]. Di-iodinated polyvinyl phenol was prepared via aromatic electrophilic substitution using sodium iodide (Figure 11B). Then, di-iodinated polyvinyl phenol was co-assembled with polystyrene-b-polyethylene glycol (PS-b-PEG) to produce micelles with iodine loadings up to 45 wt.% [46]. In addition, macromolecular ICMs poly(MAOTIB) was prepared via the free radical polymerization of MAOTIB (Figure 11C). Poly(MAOTIB) can be co-assembled with PEGylated surfactant PEG-35 castor oil [27]. The obtained micelles can be formulated with a size of about 140–200 nm, exhibiting a very strong X-ray attenuation capacity for blood pool. As shown in Figure 11D, a variety of iodinated aliphatic polyesters with high biocompatibility and biodegradability, as well as tunable thermal and mechanical properties, were prepared. Then, the co-assembly of thermosets with PEG-monostearate and lecithin was used to obtain iodinated micelles. The initial studies indicated that these micelles show good continual contrast without uptake into the kidneys [38]. These results indicate that the co-assembly strategies show great potential for the fabrication of iodinated polymer micelles for CT imaging.

**Figure 12:**
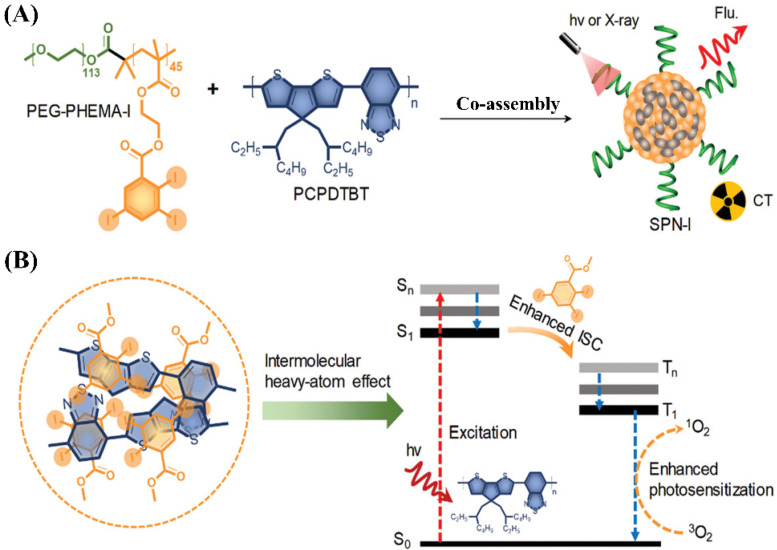
Schematic illustration of multimodal iodinated micelles for CT/fluorescence dual-modal imaging [127]. (**A**) Co-assembly of iodinated polymer PEG-PHEMA-I and semiconducting polymer PCPDTBT; (**B**) enhanced photosensitization by iodine-induced heavy-atom effect. Reprinted with permission from ref. [127], Copyright 2020 Wiley.

It is well known that each imaging modality has its own unique advantages and intrinsic limitations. Thus, a single imaging modality can no longer satisfy the rapidly growing demand for the more reliable and accurate detection of disease sites. Recently, to resolve this problem, the use of multimodal imaging, i.e., combining two or more imaging modalities into one system, has gained significant attention, because this synergistic imaging can overcome the limitations and take advantage of the strengths of each modality [1,112,113,114,115]. Co-assembly strategies have also been used to prepare multimodal iodinated micelles. For example, Zhou et al. reported novel iodine-rich semiconducting polymer-based multimodal iodinated micelles for use as contrast agents for CT and fluorescence dual-modal imaging [127]. The combination of CT and fluorescence imaging is due to the fact that the fluorescence imaging has relatively high sensitivity, which can compensate for the low sensitivity of CT imaging. Iodine-grafted amphiphilic copolymer (PEG-PHEMA-I) was firstly prepared via a combination of atom transfer radical polymerization of 2-hydroxyethyl methacrylate (HEMA) and esterification with 2,3,5-triiodobenzoic acid. Then, the semiconducting polymer (PCPDTBT) as the source of NIR fluorescence signal and the photosensitizer was used to co-assemble with PEG-PHEMA-I to form multimodal iodinated micelles in aqueous solution, as shown in Figure 12A. Multimodal iodinated micelles with sizes of about 50 nm not only have a high density of iodine to provide a high X-ray attenuation coefficient for CT imaging, but also possess a high content of PCPDTBT with high fluorescence quantum yields for fluorescence imaging. The performance of in vivo CT/fluorescence imaging for SPN-I was investigated using tumor bearing c_57_b_l/6_ male mice. After the injection of SPN-I, both CT and fluorescence signals in the tumor area were observed to gradually increase with time. These results indicated that SPN-I could passively accumulate in a tumor via the EPR effect and successfully detect a xenograft tumor both via CT and fluorescence imaging. Moreover, due to enhanced photosensitization via the iodine-induced heavy-atom effect, multimodal iodinated micelles have an improved ^1^O_2_ quantum yield, which can be used for efficient photodynamic therapy (Figure 12B). In vivo antitumor studies confirmed that photodynamic therapy achieves a significant tumor inhibition rate (98.7%).

#### 4.4.2. Polymersomes

Polymersomes, types of self-assembled vesicular structures of amphiphilic block copolymers, consist of a bilayer shell of amphiphilic copolymers and an aqueous core [128,129]. Apparently, the structures of polymersomes are different from the structure of micelles but very similar to liposomes. However, compared with liposomes, polymersomes have exhibited some significant advantages, such as higher stability in vivo circulation, tunable membrane property and versatility in chemical synthesis, which make them an attractive option for application in the encapsulation and delivery of various drugs [130,131,132].

**Figure 13:**
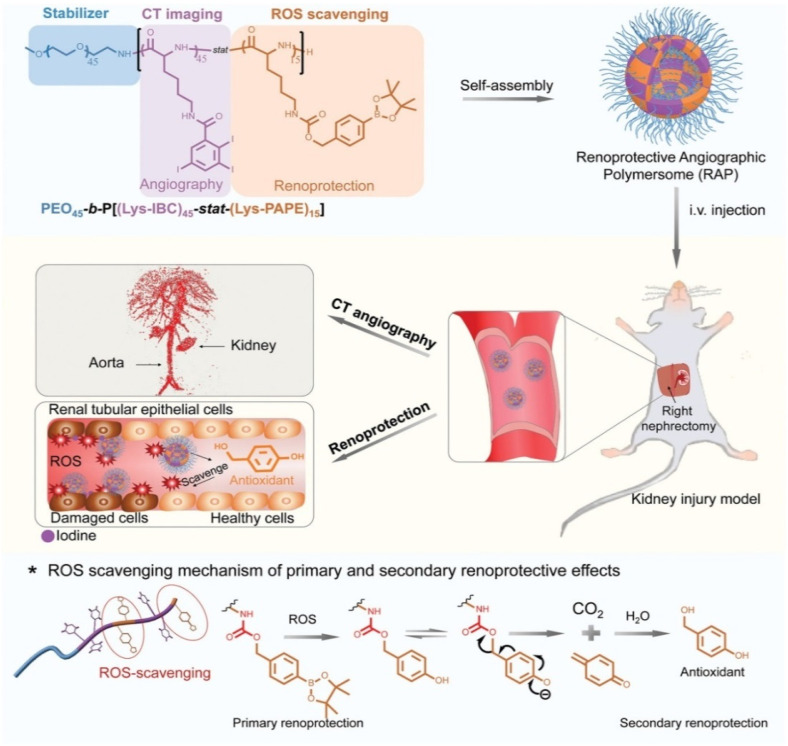
Schematic illustration of preparation process of renoprotective angiographic polymersome, the renoprotection behavior by ROS scavenging and ROS scavenging mechanism [31]. Reprinted with permission from ref. [31], Copyright 2020 Wiley.

Similar with polymer micelles, polymersomes are also not suitable for encapsulating and delivering small molecular ICMs, and no reports on this topic can be found. However, some iodine-containing amphiphilic copolymers can be assembled into iodinated polymersomes. For example, the group of Du recently designed and synthesized a kind of biodegradable, iodinated amphiphilic block copolymer poly(ethylene oxide)-block-poly(triiodobenzoic chlorideconjugated polylysine-stat-phenylboronic acid pinacol ester-conjugated polylysine) (PEO_45_-b-P[(Lys-IBC)_45_-stat-(Lys-PAPE)_15_]), which can self-assemble into renoprotective angiographic polymersomes [31], as shown in Figure 13. Polymersomes can be used as renoprotective blood pool CT contrast media due to rationally chosen repeat units. Firstly, PEO (i.e., PEG) can not only stabilize the formed polymersomes in water with a high concentration, but also endow polymersomes with a stealth function when circulating in blood. Second, Lys-IBC with high content iodine can possess a concentration-dependent X-ray attenuation capability. In addition, considering that the generation of reactive oxygen species (ROS) within the kidneys contributes to CIN pathology, Lys-PAPE with an ROS-scavenging ability was introduced into polymersomes. PAPE can scavenge ROS due to the oxidization and hydrolysis of aryl boronic ester groups. In vivo experiments indicated that the use of polymersomes as a renoprotective angiographic contrast agent can markedly reduce the risk of CIN in mice with kidney injury.

**Figure 14:**
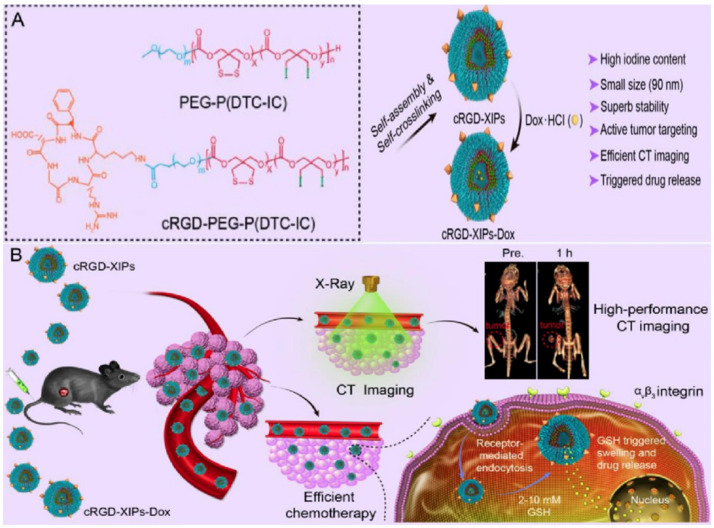
cRGD-targeted biodegradable polymersomes [133]. (**A**) Co-assembly of cRGD-PEG-b-PIC and PEG-b-PIC to prepare cRGD-targeted polymersomes (cRGD-XIPs and cRGD-XIPs-Dox); (**B**) cRGD-XIPs for enhanced in vivo CT imaging of tumor and cRGD-XIPs-Dox for targeted chemotherapy. Reprinted with permission from ref. [133], Copyright 2019 Ivyspring.

To improve tumor accumulation and retention rates, some researchers designed and developed some active targeting polymersomes. Recently, the group of Zhong designed and developed a kind of cyclic RGD-directed, disulfide crosslinked iodine-rich biodegradable polymersome (cRGD-XIP), as shown in Figure 14. The cRGD-XIPs were prepared via the co-assembly of poly(ethylene glycol)-b-poly(dithiolane trimethylene carbonate-co-iodinated trimethylene carbonate) copolymer (PEG-P(DTC-IC)) and cRGD functionalized PEG-P(DTC-IC) (cRGD-PEG-P(DTC-IC)) [133]. These novel theranostic polymersomes with size of about 90 nm exhibited a very high content of iodine (55.5 wt.%). Moreover, the cRGD-XIPs achieved a high loading content of doxorubicin (15.3 wt.%) via the pH-gradient method. Moreover, the polymersomes cRGD-XIPs and doxorubicin-loaded cRGD-XIPs (cRGD-XIPs-Dox) possessed superior colloidal stability during blood circulation due to the disulfide-crosslinked nanostructure but underwent fast drug release within tumor cells in response to the reductive microenvironment. Moreover, cRGD can act as an active targeting ligand to α_v_β_3_ integrin on overexpressed cancer cells. Thus, cRGD-targeted polymersomes can actively deliver and preferentially accumulate in tumor tissues. Compared with Iohexol, in vivo CT imaging of cRGD-XIP-treated mice presented much stronger tumor contrast. These results demonstrated that cRGD-XIPs can serve as a robust, non-toxic and smart theragnostic agent with the ability to significantly enhance CT imaging of tumors. Moreover, cRGD-XIPs-Dox displayed an enhanced targetability to tumors and achieved an elevated accumulation in tumors, which was significantly effective in inhibiting the growth of B16 melanoma model. Similarly, they also reported a kind of tumor-targeted biodegradable polymersome derived from the self-assembly of iodinated amphiphilic polyesters (cRGD-PEG-b-PIC and PEG-b-PIC), which possessed not only an ultrahigh iodine content, but also an excellent ability to target neovascular and α_v_β_3_ integrin due to the presence of cRGDfK cyclic peptide [30]. They first synthesized the iodinated amphiphilic polyester PEG-b-PIC and cRGDfK cyclic peptide (cRGD) functionalized polyester (cRGD-PEG-b-PIC) via ring-opening polymerization of a new iodine-functionalized trimethylene carbonate (IC) monomer using mPEG-OH and NHS-PEG-OH as initiator, respectively. The co-assembly of cRGD-PEG-b-PIC and PEG-b-PIC can form stable polymersomes with a small hydrodynamic size of about 100 nm. The obtained polymersomes were demonstrated to have an unprecedented iodine content (about 60 wt.%), low viscosity, and iso-osmolality, as well as long circulating property. In α_v_β_3_ integrin-overexpressing B16 melanoma xenografted mice, the cRGD-targeted polymersomes achieved a significantly higher tumor accumulation and yielded more a sufficient contrast of tumors at 6–8 h after administration when compared to Iohexol and nontargeted polymersomes groups. This kind of targeted polymersome showed great potential for application in high-performance targeted CT imaging.

**Figure 15:**
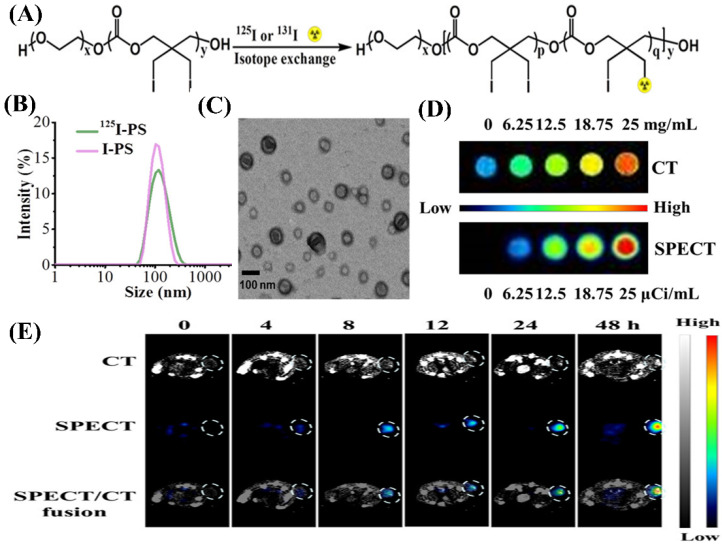
Radiolabeled iodine-rich polymersomes as multimodal contrast agent for SPECT/CT imaging [25]. (**A**) Synthesis of PEG-PIC (^125^I) and PEG-PIC (^131^I) by isotopic exchange; (**B**) size distribution profiles of I-PS and ^125^I-PS; (**C**) morphology of ^125^I-PS; (**D**) phantom reconstructions of ^125^I-PS measured at different polymer concentrations; (**E**) coronal section of in vivo CT, SPECT, and fusion images of mice. Reprinted with permission from ref. [25], Copyright 2019 American Chemical Society.

To meet the demand of high sensitivity and high-spatial resolution diagnosis of tumors, the group of Zhong recently developed a kind of iodine-rich polymersome (I-PS) and then, the I-PS was labeled with radioiodine (^125^I and ^131^I) [25], as shown in Figure 15. ^125^I and ^131^I have been demonstrated to be able to be used for imaging and radioisotope therapy, respectively. ^125^I-PS with size of about 100 nm exhibited not only a prolonged circulation, but also an obviously enhanced distribution in tumors and the reticuloendothelial system. Meanwhile, ^131^I-PS used for radioisotope therapy could significantly inhibit the growth of 4T_1_ breast tumors and effectively prolong mice survival time. More importantly, the ^125^I-labeled I-PS was demonstrated to be able to effectively achieve high-efficiency CT imaging and SPECT imaging as multimodal contrast agent for breast cancer in vivo. This study provided a robust and versatile platform for dual-modal imaging and targeted radioisotope therapy.

#### 4.4.3. Dendrimers

Dendrimers are another very important and stable nanoplatform for the development of polymer-based contrast media for use in computed tomography [134,135]. Dendrimers represent a versatile and well-defined nanoscale architecture, which are a class of unique polymeric molecules. They are synthesized in a step-wise fashion, generally starting from a multifunctional core of the dendrimer and then outwards, growing by the layer-by-layer addition of monomeric units. With the increase in the generation, the morphological structure of dendrimers will turn into a globular shape. These hyperbranched macromolecules are often monodispersed but have a highly branched and tree-like molecular architecture with uniform composition, well-defined geometry and abundant terminal functional groups. Especially, abundant terminal functional groups can not only provide many sites for functionalization at the ends of the branches, but also offer many reactive groups for the conjugation of drugs at the available termini of the molecules. Compared with linear polymers, dendrimers often have a higher solubility in various solvents and show much lower viscosity under the same conditions. In addition to their nanometric size range, permeability across the biological membrane and a relatively high biocompatibility, dendrimers have displayed significant potential as a versatile delivery system for drugs and diagnostic agents [69,136,137].

**Figure 16:**
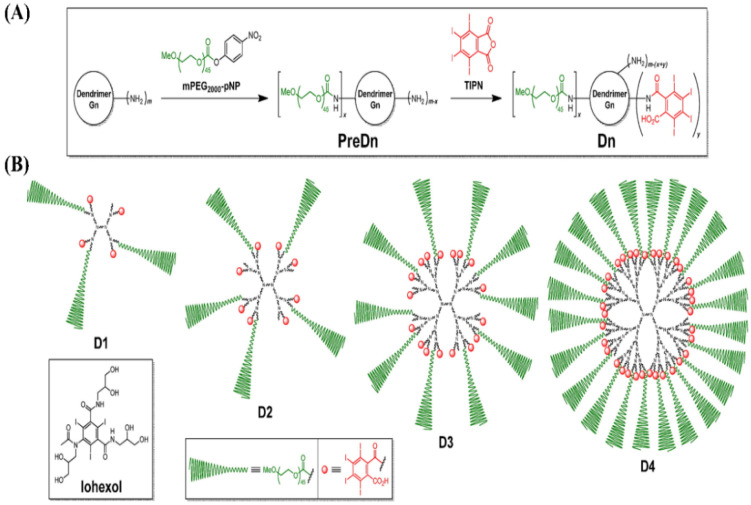
Synthesis process of iodinated dendritic nanoparticles [56]. (**A**) Synthetic process; (**B**) dendritic CT contrast agents. Reprinted with permission from ref. [56], Copyright 2016 Elsevier.

As a typical dendrimer, poly(amido amine) (PAMAM) consists of an ethylenediamine core, tertiary amine branches, and alkyl amide spacers, which allows for functionalization and drug conjugation through amine groups on its outer surface. PAMAM has been widely used to load and deliver ICMs to increase imaging time, decrease rental toxicity and improve specificity. For example, amine-terminated fourth-generation (G4) PAMAM dendrimers were used as a multifunctional platform to conjugate a small iodinated compound 3-N-[(N,N-dimethylaminoacetyl) amino]-a-ethyl-2,4,6-triiodobenzenepropanoic acid. The obtained iodinated dendritic nanoparticles [G-4-(DMAA-IPA)_37_] with a hydrodynamic radius of 2.4 nm can achieve 33% iodine content by weight and retain their high water solubility [138]. Amine-terminated third- and fourth-generation PAMAM dendrimers with ethylenediamine cores were also conjugated with tetraiodobenzene derivatives to prepare blood pool contrast media for use in CT imaging [56], as shown in Figure 16. The obtained unimolecular dendritic contrast media with the size of 13–22 nm are water soluble and exhibited high contrast enhancement in the blood pool and effectively extended their blood half-lives. Fu et al. synthesized a series of paired, symmetrical dendritic polylysines initiated from a large PEG core (3000–12,000 g/mol). Then, triiodophthalamide molecules were conjugated onto the amine termini of these dendrimers. The in vivo enhancement for CT contrast in a rat model was evaluated. The results indicated that the iodinated PEG-core dendrimer conjugates achieve high X-ray attenuation intensity, high water solubility, good chemical stability and persistent intravascular enhancement, with a blood half-life of about 35 min [55].

#### 4.4.4. Polymeric Solid Nanoparticles

Although polymeric micelles and polymersomes have been demonstrated to have significant potential for improving the delivery efficiency and circulation time of ICMs, their intrinsic instability limits their wider applications [32,33,126]. Therefore, developing polymeric NPs with high stabilities is highly demanded. Polymeric solid nanoparticles (SNPs) with stable core structures due to crosslinking or multiple interactions can serve as an excellent platform for ICMs delivery. For example, some core-crosslinked polymeric SNPs were designed and developed for the delivery of contrast agents. Ding et al. reported a one-pot strategy for the synthesis of core-crosslinked Iohexol nanoparticles (INPs) on a large scale for CT imaging [139]. They used Iohexol acrylate as a crosslinking agent via polymerization-induced self-assembly to achieve the high stability and good dispersion of INPs even in an extremely high concentration. INPs can not only have lower toxicity and a longer circulation time, but also exhibit strong imaging capability and prominent accumulation in tumors when compared with Iohexol. Hainfeld et al. reported a kind of PEG-coated core-crosslinked polymer iodinated nanoparticle with a size of about 20 nm [140]. Iodine SNPs are a polymerized triiodobenzene compound coated with PEG, which were demonstrated to not only have an extraordinarily long blood half-life (40 h) for better tumor uptake, but to also be non-toxic after an intravenous dose of 4 g iodine/kg. These iodine SNPs may serve as an X-ray contrast agent with novel properties for cancer therapy and vascular imaging.

**Figure 17:**
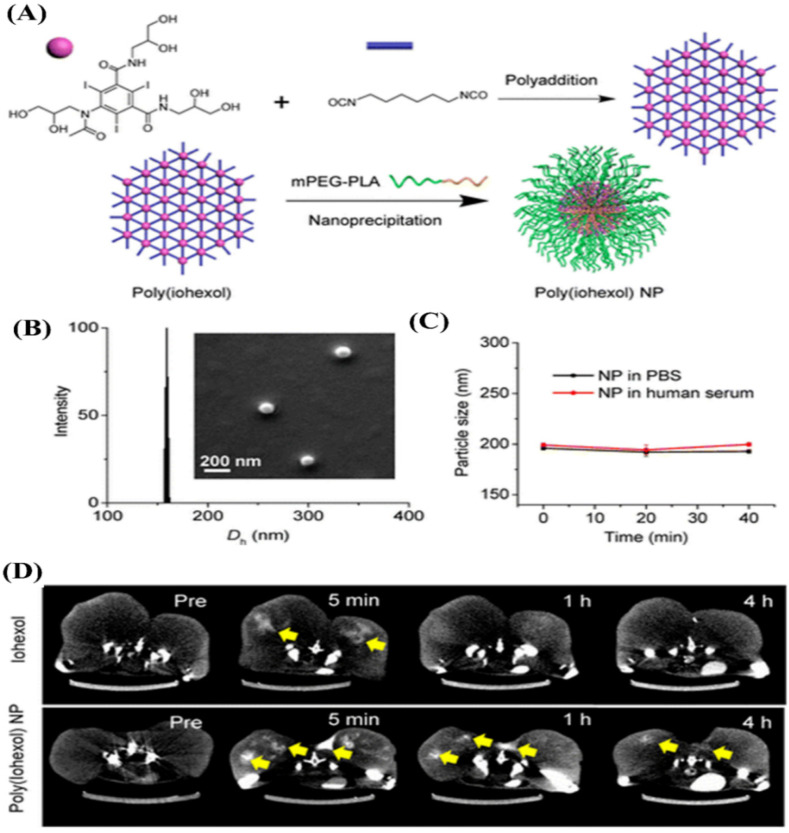
Synthesis and structure of core-crosslinked poly(iohexol) SNPs [141]. (**A**) Synthetic illustration of preparing cross-linked poly(iohexol) and PEGylated poly(iohexol) SNPs; (**B**) size and morphology; (**C**) stability in PBS or human serum buffer for different times; (**D**) serial axial CT images of the MCF-7 tumors in mice after intratumoral injection Iohexol and poly(iohexol) SNPs. Reprinted with permission from ref. [141], Copyright 2013 American Chemical Society.

Similarly, the Cheng group developed a kind of poly(iohexol) SNP by using the addition reaction between hexamethylene diisocyanate and Iohexol with multiple hydroxyl groups as a comonomer [141], as shown in Figure 17A. After nanoprecipitation with mPEG-polylactide (mPEG-PLA), poly(iohexol) SNPs with sizes of about 150 nm in diameter with narrow size distributions were obtained (Figure 17B). PEGylated poly(iohexol) SNPs exhibited remarkable stability without any significant size changes or premature release of Iohexol in PBS and human serum buffer, as the crosslinked core could prevent disassembly against dilutions upon administration (Figure 17C). The potential of poly(iohexol) SNPs for in vivo CT diagnosis was evaluated and is shown in Figure 17D. The results indicated that poly(iohexol) SNPs with high stability exhibited a substantial improvement in tissue retention and CT contrast (a 36-fold increase in CT contrast 4 h post injection).

Considering their endogenous origin, nonimmunogenic, biocompatible and biodegradable nature as well as relatively high stability, lipoproteins, including low-density lipoproteins (LDL) and high-density lipoproteins (HDL) as natural SNPs, have been demonstrated to be highly suitable as a platform for delivering imaging agents [28,75,142]. For example, the radio-iodine as radiotracers, including iodine-131 (^131^I) and iodine-125 (^125^I), were used to label LDL. The obtained radio-iodinated LDL SNPs were used to image and characterize tumor accumulation within animals for over several decades [143]. Zheng et al. incorporated poly-iodinated triglyceride into LDL for the delivery of a CT contrast agent [144], achieving an enhancement on CT imaging via LDL-induced RES targeting. Radiopaque iodinated copolymeric SNPs with sizes ranging between 30 and 350 nm were prepared via the emulsion copolymerization of MAOETIB and glycidyl methacrylate (GMA) in the presence of sodium dodecyl sulfate as a surfactant and potassium persulfate as an initiator. The obtained P(MAOETIB-GMA) SNPs with high iodine contents of 58% possess a significant radiopaque nature. In vivo CT imaging was performed in a dog model. The results indicated that the obtained P(MAOETIB-GMA) SNPs can achieve significant enhanced visibility of the liver, spleen and lymph nodes of model animals by RES-selective uptake [43].

**Figure 18:**
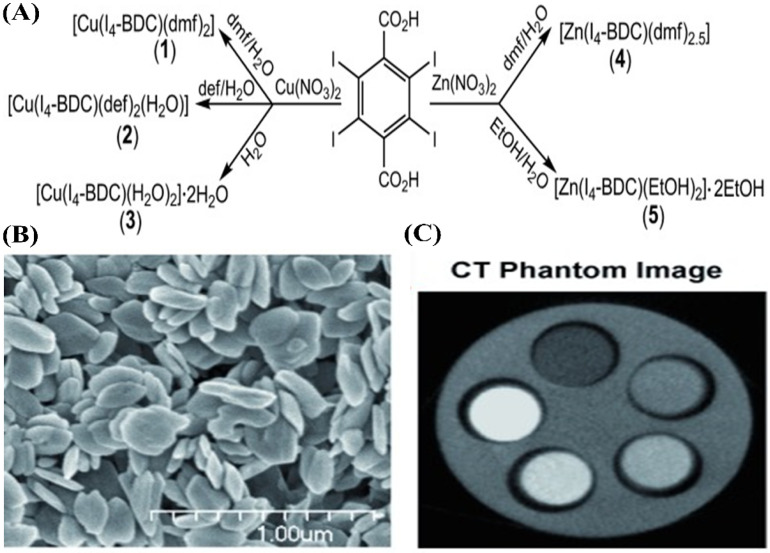
Iodinated coordination polymer SNPs with high payload of iodine [145]. (**A**) Synthesis process; (**B**) Typical morphology of iodinated coordination polymer SNPs; (**C**) CT phantom images. Reprinted with permission from ref. [145], Copyright 2009 Wiley.

Recently, Krafft et al. reported a series of stable iodinated coordination polymer SNPs with the ability to carry a very high payload of iodine (over 60 wt.%) [145]. As shown in Figure 18A, 2,3,5,6-tetraiodo-1,4-benzenedicarboxylic acid (I_4_-BDC-H_2_) as bridging ligands and CuII or ZnII metal as connecting points were used to synthesize five new coordination polymer SNPs. Scanning electron microscopy images confirmed that the formed iodinated coordination polymer SNPs, typically polymer (3), are plate-like particles, 50 nm thick and with a diameter of 300 nm and (Figure 18B). This is due to the fact that each CuII center can coordinate to two water molecules and three carboxylate oxygen atoms in a square pyramidal geometry. They also conducted phantom studies on the obtained polymer SNPs to evaluate their potential for use as CT contrast media. As shown in Figure 18C, the coordination polymer SNPs show a very high X-ray attenuation coefficient, which can be comparable to that of the molecular contrast agent (Iodixanol). These new nanomaterials can deliver high payloads of iodine, which shows that they have great potential for the development of efficient CT contrast media without the inherent drawbacks of small-molecule ICMs.

**Figure 19:**
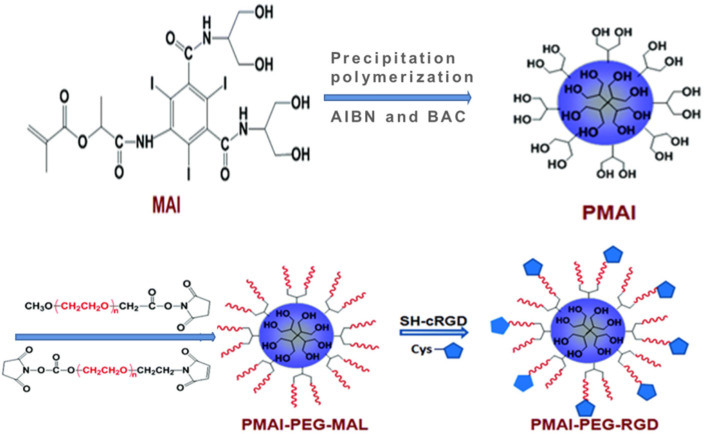
Synthesis process of poly(methacrylated iopamidol)-polyethylene glycol-cRGD (PMAI-PEG-RGD) SNPs [146]. Reprinted with permission from ref. [146], Copyright 2020 Royal Society of Chemistry.

To further achieve targeted CT imaging on tumors, Gao et al. firstly synthesized a kind of methacrylated Iopamidol (MAI) monomer, and then, MAI was polymerized to obtain poly(methacrylated Iopamidol) (PMAI) SNPs via a precipitation polymerization method [146], as shown in Figure 19. Subsequently, PMAI SNPs were PEGylated via the introduction of PEG chains, and then, the targeting ligand cRGD peptide was conjugated onto the outer surface to obtain poly(methacrylated iopamidol)-polyethylene glycol-cRGD (PMAI-PEG-RGD) SNPs with sizes of about 150 nm and iodine contents of about 30 wt.%. The X-ray attenuation capability of PMAI-PEG-RGD SNPs was detected. Compared with Iopamidol, the stronger X-ray attenuation effect of PMAI-PEG-RGD SNPs was demonstrated. These results indicated that PMAI-PEG-RGD SNPs can act as a promising contrast agent for X-ray CT imaging. More importantly, PMAI-PEG-RGD SNPs were endowed with tumor-targeting ability due to the presence of cRGD ligand with a specific affinity for α_ν_β_3_ integrin overexpressed on cancer cells. In vivo CT imaging indicated that PMAI-PEG-RGD SNPs can show greatly enhanced CT imaging efficacy, confirming their more efficient tumor accumulation due to the cRGD peptide-mediated active targeting effect.

Hyaluronic acid (HA), as a highly water-soluble, negatively charged polysaccharide, was widely used to increase stability in aqueous solution and prolong the circulation time of nanoparticles in vivo. More importantly, HA, as a specific ligand for CD44 often overexpressed on tumor cells, was also applied for the tumor-targeted delivery of anticancer drugs and imaging contrast media [147]. Liu et al. recently reported a facile but effective approach to synthesize multifunctional HA-coated iodinated SNPs with Au nanoshells (PMATIB/PEI/Au nanoshell/HA) [26]. They first prepared iodinated crosslinked SNPs (PMATIB) via the precipitation polymerization of 2-methacryl(3-amide-2,4,6-triiodobenzoic acid) (MATIB) using N,N-methylenebis-(acrylamide) (MBAAm) as a cross-linker. Subsequently, PMATIB SNPs were modified with PEI and ultrafine Au NPs through the electrostatic interaction. Finally, HA was coated on the outer surface to obtain PMATIB/PEI/Au nanoshell/HA SNPs with sizes of about 200 nm and excellent dispersibility in aqueous solution. After intravenous injection into MCF-7 tumor-bearing mice, PMATIB/PEI/Au nanoshell/HA SNPs could efficiently be accumulated in the tumor and significantly enhance CT imaging of the tumor. Pan et al. presented a novel approach based on a soft, radio-opaque, and vascular-constrained colloidal particle, which can improve targeting specificity for intraluminal thrombus [148]. In this study, the amphiphilic diblock copolymer, polystyrene-b-polyacrylic acid (PS-b-PAA) was used to encapsulate ethiodized oil (a mixture of iodostearic acid ethyl ester and ethyldiiodostearate with 37 wt.% of total iodine content). Then, the particles were crosslinked via carbodiimide-mediated intramolecular cross-linking and then conjugated with biotin hydrazide as the targeting ligand on the surface of cross-linked particle, which finally obtained the soft type, vascularly constrained, stable colloidal radio-opaque iodinated polymeric SNPs (iodinated-cROMP-Biotin). The carboxylic acid groups throughout the nanoparticle shell led to a significant enhancement on stability. Moreover, the biotinylated cROMP particles were demonstrated to be able to effectively target to acellular fibrin clot phantoms with classic avidin–biotin interactions.

Single CT imaging modality often cannot satisfy the rapidly growing demand for the more reliable and accurate detection of disease sites, due to its low sensitivity. The combination of two or more imaging modalities into one system can overcome the limitations and take advantage of the strengths of each modality [1,112,113,114,115]. Polymeric SNPs were also designed for multimodal imaging. For example, the group of Whittaker designed and synthesized a kind of multifunctional, crosslinked hyperbranched polymer SNP containing iodine and fluorine, which can be used as bimodal imaging contrast media for use in CT/19F MRI imaging [149]. The hyperbranched iodopolymer (HBIP) was first synthesized via the reversible addition–fragmentation chain transfer polymerization of poly(ethylene glycol) methyl ether methacrylate (PEGMA), 2-(2′,3′,5′-triiodobenzoyl)ethyl methacrylate (TIBMA), and a degradable crosslinker bis-(2-methacryloyl)oxyethyl disulfide (DSDMA). Then, hyperbranched iodopolymers containing 19F (HBIPFs) with different contents of iodine and fluorine were prepared via the chain-extension reaction between the HBIP with PEGMA and 2,2,2-trifluoroethyl acrylate (TFEA). After the direct dissolution of HBIPFs in water, HBIPF SNPs with diameters of 10-15 nm were obtained. The radio-opacity of HBIPF SNPs in water was investigated using 19F MRI and CT imaging. The results indicated that HBIPF SNPs are attractive multimodal imaging contrast media for use in CT/19F MRI bimodal imaging.

Due to its high safety, low cost and portability, ultrasound (US) imaging has been widely utilized in clinical diagnosis. However, US imaging often suffers from very low resolution. The combination of high-resolution CT imaging with US imaging has significant merits for the development of multimodal imaging. To obtain real-time imaging and additional anatomic information about a tumor, Choi et al. synthesized a kind of iodine containing diatrizoic acid (DTA)-conjugated glycol chitosan (GC) SNP, which was used to physically encapsulate a US imaging agent (perfluoropentane, PFP) via the O/W emulsion method to prepare GC-DTA-PFP nanoparticles [59]. The in vitro and in vivo X-ray CT/US dual-modal imaging efficacy of GC-DTA-PFP SNPs was evaluated. The results indicated that as imaging contrast agents, GC-DTA-PFP SNPs presented very strong X-ray CT and US signals in phantom tests. Moreover, after intravenous injection, GC-DTA-PFP SNPs can be effectively accumulated on the tumor site by EPR effects, which thus could be used in X-ray CT/US dual-modal imaging to provide comprehensive and accurate diagnostic information about a tumor. In sum, due to their high stability, excellent biocompatibility, multifunctionality and flexibility in modification, polymeric SNPs provide a variety of multifunctional platforms for not only improved ICMs delivery, but also the development of multimodal contrast media for multimodal imaging.

**Figure 20:**
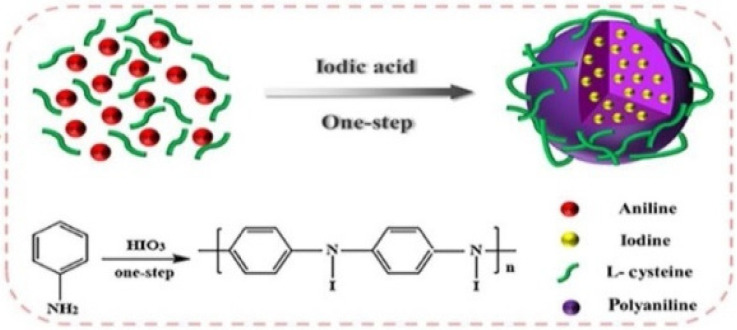
Schematic illustration of the preparation process of LC@I-PANi SNPs as multimodal contrast agent for CT/PA dual-modal imaging [48]. Reprinted with permission from ref. [48], Copyright 2021 Elsevier.

Photoacoustic (PA) imaging is a type of biomedical imaging based on laser-generated ultrasound. As a new and hybrid modality, PA imaging integrates the high spatial resolution and deep penetration of ultrasound imaging with the high-contrast and specificity of optical imaging [150]. Polyaniline (PANi), with intense near-infrared (NIR) absorbance and a stable light-to-heat conversion capacity, has exhibited excellent imaging capability as a PA contrast agent [151]. To design multimodal contrast media for CT/PA-guided therapy, recently, Fu et al. rationally designed and developed a kind of iodinated polyaniline (LC@I-PANi) SNP via the simultaneous iodination and chemical oxidation polymerization of aniline in one system [48], as shown in Figure 20. LC@I-PANi SNPs with sphere-like morphologies and around 170 nm diameters have excellent colloidal stability and high biocompatibility. Furthermore, in vitro and in vivo experiments confirmed that LC@I-PANi SNPs possess favorable CT and PA imaging performance and good photothermal performance under NIR laser irradiation, providing a promising multifunctional therapeutic nanoplatform.

## 5. Conclusions

The clinical applications of X-ray CT imaging in medical diagnosis are still limited by the intrinsic drawbacks of iodine-based contrast media. Small molecular ICMs, used as the main contrast media in clinic, still suffer from fast renal clearance and serious adverse effects, especially the acute renal toxicity and inefficient targetability as well as low sensitivity. Due to their unique advantages, organic NPs, especially polymeric NPs, have exhibited great potential for the development of next-generation drug delivery systems with desirable properties. In this review, we comprehensively summarized the strategies and applications of organic NPs for ICMs delivery. Undoubtedly, these nanocarriers can significantly prolong blood circulation time, decrease renal toxicity, enhance delivery targetability and improve the sensitivity.

Despite the tremendous progress, the use of organic NPs for ICMs delivery is still far from applicable to clinical practice at the moment. Many challenges, such as batch-to-batch reproducibility, long-term biocompatibility, specific delivery, in vitro/in vivo stability and desirable pharmacokinetics need to be urgently overcome. As a result, tremendous efforts are still needed to develop efficient organic NPs for ICMs delivery. First, the scale-up preparation of organic NPs with controlled and uniform morphology is still a big challenge. With regard to nanomedicines and nanoimaging agents, the uniformity in nanostructure, the stability in physicochemical property and therapeutic performance, especially the controllability in the preparation process and the reproducibility in product quality are very important requirements for pharmaceutical and biomedical applications. However, due to the heterogeneity in the raw materials and the complexity and extremely high variability in the preparation process, the biomedical applications of organic NPs need to address the scalable production and batch-to-batch reproducibility. Thus, it is imperative to develop an effective approach to fabricate organic NPs with precise sizes, nanostructures and geometries in a scalable process, achieving high uniformity, reproducibility and thus high-performance. Second, ICMs were loaded into various organic NPs, often leading to a relatively low iodine content. As a result, a massive dose of contrast agents often necessitates the need for clear CT imaging, which will always pose a risk of renal toxicity and cytotoxicity. In addition, the in vivo degradation behaviors or decomposition product of organic NPs remain unclear, and thus thorough toxicological evaluations will be needed to confirm the biocompatibility of organic NPs for ICMs delivery. In addition, the delivery of sufficient amounts of contrast media in the targeted disease site is indispensable for successful imaging. Therefore, how to avoid fast clearance from the system but achieve the high accumulation of ICMs in malignant tissues is a key issue yet to be resolved, especially for the preoperative and intraoperative identification of tumors as well as intraoperative image-guided surgery. Significant efforts have been focused on improving the ability to target the delivery of ICMs via use of active and passive targeting strategies. However, the sophisticated pathophysiological barriers from the injection site to the site of action and the unsatisfying targetability of nanocarriers result in a very low delivery efficiency. Despite the enormous progress in nanomedicines, the design and development of advanced nanocarriers that can simultaneously meet the contradictory requirements to successively overcome each of the biological barriers is still a key issue to be addressed. Finally, we are witnessing a paradigm shift from conventional therapy to a more personalized, customized treatment model based on theragnosis. As a result, theragnostic agents must synergistically integrate multiple functions, including the therapeutic efficacy of drugs, disease recognition via imaging and targeted delivery to disease sites, leading to a formidable challenge in the fabrication of theragnostic agents. Organic NPs have been demonstrated to be a promising platform for theragnostic agents but are still in their infancy.

In sum, various organic NPs have exhibited significant advantages for ICMs delivery, opening up some new avenues in the search for optimal ICMs for use in CT imaging with maximum sensitivity, minimal toxicity, improved specificity and biodistribution.

## Data Availability

Not applicable.
